# “Indus Kay Sitaray”: A novel hospital-based school at The Indus Hospital, Karachi

**DOI:** 10.12669/pjms.36.ICON-Suppl.1727

**Published:** 2020-01

**Authors:** Tooba Nadeem Akhtar, Muhammad Fareeduddin, Muhammad Shamvil Ashraf

**Affiliations:** 1 Child Life Counselor, The Indus Hospital, Karachi, Pakistan; 2 HOD, Pediatrics, The Indus Hospital, Karachi, Pakistan; 3 Executive Director Medical Services, The Indus Hospital, Karachi, Pakistan

The Indus Hospital (TIH), Karachi, diagnoses over 150 children with cancer and other chronic diseases monthly. The impact of this is weeks spent away from classrooms. School absenteeism due to chronic disease leads a multitude of problems pertaining to emotional, social and cognitive development and behaviors, and vulnerability to stress or secondary illnesses.^1^

To address this gap, the Departments of Pediatric Medicine and Psycho-Social Care at TIH teamed with Shah Wilayat Public School (SWiPS) to form ‘**Indus Kay Sitaray’ (IKS)** – Pakistan’s first hospital schooling program.

Since its inception, IKS has completed two academic sessions with nearly 30 elementary school graduates. Graduates gain certification following final examination by SWiPS and a letter facilitating their admission into mainstream schools upon completion of medical treatment.

Hospital-schools provide a familiar and comforting routine to children with chronic illness. With research currently underway, counselors have observed patients to appear less withdrawn during treatment and have a higher pain tolerance during invasive procedures like intravenous cannulation and intrathecal chemotherapy. A hospital schooling program is an effective method of addressing the psychosocial needs of an entire family unit.^2^-^4^ In the next phase, we hope to involve parents in literacy classes.

Our experience at IKS can provide useful learning for those interested in implementing hospital-schooling programs. For more information, please email at induskaysitaray@tih.org.pk.
